# COVID-19 Vaccination Acceptance and Hesitancy among Healthcare Workers in Germany

**DOI:** 10.3390/vaccines9070777

**Published:** 2021-07-12

**Authors:** Christopher Holzmann-Littig, Matthias Christoph Braunisch, Peter Kranke, Maria Popp, Christian Seeber, Falk Fichtner, Bianca Littig, Javier Carbajo-Lozoya, Christine Allwang, Tamara Frank, Joerg Johannes Meerpohl, Bernhard Haller, Christoph Schmaderer

**Affiliations:** 1 Department of Nephrology, School of Medicine, Technical University of Munich, Klinikum Rechts der Isar, 81675 Munich, Germany; matthias.braunisch@tum.de (M.C.B.); bianca.littig@web.de (B.L.); javier.carbajolozoya@mri.tum.de (J.C.-L.); Christoph.Schmaderer@mri.tum.de (C.S.); 2 TUM Medical Education Center, School of Medicine, Technical University of Munich, 81675 Munich, Germany; 3 Department of Anesthesiology, Intensive Care, Emergency Medicine and Pain Medicine, University Hospital Wuerzburg, 97080 Wuerzburg, Germany; Kranke_P@ukw.de (P.K.); popp_m4@ukw.de (M.P.); 4 Department of Anesthesiology and Intensive Care, University Hospital Leipzig, 04103 Leipzig, Germany; christian.seeber@medizin.uni-leipzig.de (C.S.); falk.fichtner@medizin.uni-leipzig.de (F.F.); 5 School of Medicine, Technical University of Munich, Klinikum Rechts der Isar, Clinic and Polyclinic for Psychosomatic Medicine and Psychotherapy, 81675 Munich, Germany; christine.allwang@mri.tum.de (C.A.); tamara.frank@mri.tum.de (T.F.); 6 Medical Center & Faculty of Medicine, Institute for Evidence in Medicine, University of Freiburg, 79110 Freiburg, Germany; meerpohl@ifem.uni-freiburg.de; 7 Cochrane Germany, Cochrane Germany Foundation, 79110 Freiburg, Germany; 8 Institute of Medical Informatics, School of Medicine, Technical University of Munich, Statistics and Epidemiology, 81675 Munich, Germany; bernhard.haller@mri.tum.de

**Keywords:** COVID-19, vaccine, vaccination, vaccination hesitancy, vaccine refusal, vaccination campaign

## Abstract

Vaccination hesitancy is a threat to herd immunity. Healthcare workers (HCWs) play a key role in promoting Coronavirus disease 2019 (COVID-19) vaccination in the general population. We therefore aimed to provide data on COVID-19 vaccination acceptance/hesitancy among German HCWs. For this exploratory, cross-sectional study, an online survey was conducted in February 2021. The survey included 54 items on demographics; previous vaccination behavior; trust in vaccines, physicians, the pharmaceutical industry and health politics; fear of adverse effects; assumptions regarding the consequences of COVID-19; knowledge about vaccines; and information seeking behavior. Odds ratios with 95% confidence intervals were calculated and chi-square tests were performed. Four thousand five hundred surveys were analyzed. The overall vaccination acceptance was 91.7%. The age group ≤20 years showed the lowest vaccination acceptance. Factors associated with vaccination hesitancy were lack of trust in authorities and pharmaceutical companies. Attitudes among acquaintances were associated with vaccination hesitancy too. Participants with vaccination hesitancy more often obtained information about COVID-19 vaccines via messenger services or online video platforms and underperformed in the knowledge test. We found high acceptance amongst German HCWs. Several factors associated with vaccination hesitancy were identified which could be targeted in HCW vaccination campaigns.

## 1. Introduction

The coronavirus disease 2019 (COVID-19) pandemic is one of the biggest healthcare challenges in history. It has been estimated that a pandemic spread could be stopped if more than 67% of the population acquire immunity by either vaccination or infection [1]. Allowing uncontrolled infection would cause increased morbidity and mortality and an unjustifiable strain on healthcare systems [2]. Furthermore, even mild to moderate COVID-19 infections might lead to long-term health consequences [3] and individual financial losses [4]. Therefore, large scale population-wide vaccination programs are the preferred approach to stop the pandemic. However, vaccine hesitancy is a global barrier to this strategy and is rated among the top ten threats worldwide [5,6].

Herd immunity cannot be achieved if pronounced vaccine hesitancy is present [7]. Healthcare workers (HCWs) have an important advisory role in vaccination programs, as they are required to provide information and thus build confidence. Additionally, HCWs who are at risk of acquiring infections from their patients are often regarded as the sources of nosocomial infections with vaccine-preventable diseases [8], a circumstance that appears particularly threatening during the COVID-19 pandemic. Therefore, COVID-19 vaccine acceptance in HCWs is of high importance, and reasons for hesitancy need to be assessed. In a representative national survey in Germany, the reported COVID-19 vaccination acceptance was only 66% in the general population (having risen from 50% in December 2020) and 64% in a small subset of HCWs [9] (Table S1). During our survey, infection rates (ranging from 83/10,000 in the beginning to 57/10,000 in the middle and to 64/10,000 at the end of February in Germany) [10] were lower than in December 2020 by up to 200/100,000. Recent, partially not-yet-peer-reviewed surveys of HCWs have shown vaccine acceptance rates between 31% and 86% [11,12,13,14,15,16,17,18,19,20,21,22,23,24,25,26,27] (Table S1). However, most of these studies had lower numbers of participants, were performed in single working groups or had fewer items, thereby providing descriptions of reasons for hesitancy that were lacking in detail. Associated factors mainly were lack of trust in new vaccines, efficacy and safety; fear of side effects; not feeling well informed; safety concerns; and the velocity of the development process. Additionally, only a few studies from Germany existed when we began, and they did not cover large numbers of HCWs from all German regions [11,12,13,14,15,16,17,18,19,20,21,22,23,24,25,26,27].

To design specific vaccination campaigns addressing the concerns of potential vaccine candidates in the healthcare system, sufficient data reflecting the reasons for hesitancy are necessary [28]. In the context of an ongoing pandemic caused by a new infectious disease, previous findings on vaccine hesitancy for classical vector-based vaccines such as annual influenza vaccines might not be completely applicable to COVID-19 vaccines. This is even more understandable, since the new technology of mRNA-based vaccines are being used for the first time. The individual’s decision to receive a COVID-19 vaccination is multifactorial, being based, among other things, on an individually perceived health risk, experiences with past vaccinations and sociodemographic factors [29].

The main goals of this survey in HCW were (A) to provide current numbers of HCW COVID-19 vaccination acceptance in Germany, and (B) to identify factors associated with vaccination hesitancy.

## 2. Materials and Methods

### 2.1. Study Setting and Participants

In order to conduct COVID-19 research involving German healthcare professionals, politicians, stakeholders and laity, CEOsys (COVID-19 evidence ecosystem, www.covid-evidenz.de (accessed on 1 April 2021) [30], a research group within the network of German university hospitals (NUM, Netzwerk Universitätsmedizin) was launched in 2020. The network is funded by the German Federal Ministry of Education and Research (Bundesministerium für Bildung und Forschung, BMBF). This study was conducted as part of the evaluation of informational needs.

This cross-sectional exploratory [31] study was based on an online survey conducted from 2 February 2021 to 28 February 2021 in German language. At that point of time, the vaccines Comirnaty^®^ by BioNTech/Pfizer and Vaxzevria^®^ by Astra Zeneca had been approved and were in use in Germany. The vaccination campaign had been started approximately one month before the start of this survey.

### 2.2. Study Size

A link to this voluntary open online survey was sent to a total of 3924 email addresses of nursing homes, medical practices, ambulance services, medical universities, hospitals, ambulatory care services and medical societies across Germany. The number of invitations per region and federal state can be seen in Table S2.

### 2.3. Survey Instrument

For data protection reasons, only email addresses that did not contain any personally identifying features (e.g., name of the addressee) were used. The existence of such an anonymous email address was chosen as the criterion for the random selection of the facilities. The addresses were selected from publicly accessible registries such as hospital registries, online telephone books and online physician registries for each HCW group in each German state (stratified sample [32]). As no openly accessible all German general medical registry exists, local/federal state online registries were searched. For doctor’s practices, the directories were searched for all available specialties. All anonymous email addresses of physicians’ practices, physicians’ associations and medical societies (with member numbers ranging from a few hundred to several thousand [33,34]), and all medical faculties and all emergency services with anonymous email addresses provided online that we found were contacted. In non-medical faculty hospitals, the first five hospitals with an anonymous board email address were contacted in each of the sixteen federal states. In outpatient care services, a maximum of 50 and in nursing homes a maximum of 40 anonymous email addresses per federal state were chosen (the first 50/40 with anonymous email addresses). In the invitation email, the subject was asked to forward the link within the respective institution or society (snowball sampling [32]). No further advertising was carried out by the authors. There were no incentives offered for taking part in the survey. The survey was carried out on the online platform SoSci Survey [35]. The items were not randomized. To guarantee full anonymity, no cookies were used to identify unique users, no IP checks or logfile analyses were performed and users were not asked for registration. There was no review step for the respondents.

To formulate questions, the GESIS (Leibniz Institute for the Social Sciences) survey guidelines for question wording were considered [36]. The recommendations of the Working Group on Vaccine Hesitancy Determinants Matrix [37] were applied. The matrix included contextual influences, individual and group influences and vaccine/vaccination-specific issues. In addition, the 5C-model by Betsch et al. was taken into account when constructing the questions [38]. This model includes the categories “confidence, constraints, complacency, calculation and collective responsibility”, which correlate—among others—with “attitude, self-control, perceived personal health status and invulnerability, preference for deliberation and communal orientation” [38]. Furthermore, questions were adapted from the surveys by Larson et al. [39]. Additionally, questions addressing specific previous results on vaccine hesitancy were included [40]. The questions were then discussed in detail with a sociologist (B.L.).

The survey contained 54 items (12 screens). Demographic data, previous vaccination behavior, trust in vaccines/physicians/pharmaceutical industry/health politics, fear of adverse effects, assumptions about the consequences of the disease, knowledge about vaccines and information seeking behavior of the participants were queried. Four items on the participants’ knowledge on COVID-19 vaccinations were included as well. The full questionnaire including answer options can be found in Table S3.

A survey pretest was performed by 17 members of the authors’ departments and of the CEOsys network. The questions were then revised according to their comments. Testers reported a completion time of approximately ten minutes, which was included in the study information.

We followed the Checklist for Reporting Results of Internet E-Surveys (CHERRIES) [41] (Table S4). In the item “sources of information”, a maximum of five answer options could be selected to identify the media that were essential to the participant. Questionnaires which had not been completed (i.e., which had been abandoned), questionnaires without informed consent and questionnaires with missing information on vaccination willingness/hesitancy were excluded from statistical analyses. In several questions, the answer option “no answer” was given to comply with data protection guidelines on the one hand and to avoid abandoning of the questionnaire on the other.

### 2.4. Data Collection

Data were collected using SoSci Survey [35]. Extraction of data was performed after the completion of the survey period. The data were extracted as a Microsoft Excel^®^ dataset from the “SoSci Survey” [35] survey instrument.

### 2.5. Statistical Methods

**Figure 1:**
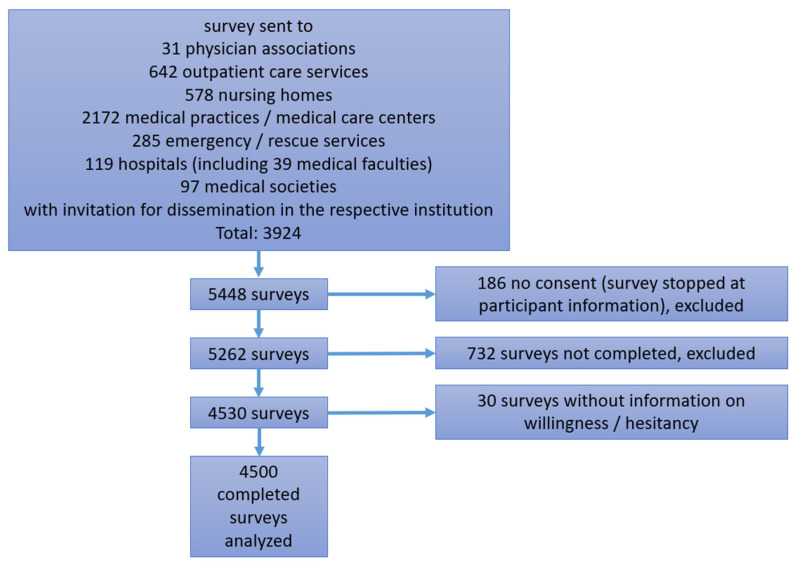
Flowchart of participants.

If a respondent replied to the questions on their professional groups and work settings with “other”, he was asked to fill in a comment to indicate his profession and work setting. These comments were analyzed, and participants were assigned to predefined professional groups where possible. The categories “dental assisting personnel”, “dentist” and “science” were newly built from the comments. For statistical analysis, the combined professional group “other non-physician medical staff” was built from the categories “non-examined nurse”, “medical specialist”, “non-physician staff in the rescue service” and “trainee.” The combined group “physicians with specialist/personnel responsibility” was built from the groups “specialized physician”, “consultant physician” and “chief physician.” Questionnaires which had not been completed (i.e., which had been abandoned), questionnaires without informed consent and questionnaires with missing information for the items of vaccination willingness/hesitancy were excluded from statistical analyses (Figure 1). For the other items, the frequencies of “no answer” are indicated in the respective tables (Tables S2 and S5) in the Supplementary Materials. These participants were not included in the statistical tests. Unless otherwise specified, participants were summarized to be “willing/accepting” if they wanted to get or had already been vaccinated. Similarly, participants who indicated they were undecided or unwilling were summarized as hesitant. German regions were defined according to the classification used by the Robert Koch Institute [42]. Statistical analysis was performed using R, version 4.0.4 (R Foundation for statistical Computing, Vienna, Austria), and its libraries “*epitools*”, “*arsenal*”, “*sf*” and “*mapplots*”.

Data are presented as absolute and relative frequencies. For group comparisons, odds ratios with corresponding 95% confidence intervals are presented, and chi-square tests were performed. All tests were two-sided and a significance level of 5% was used. Due to the exploratory nature of the study, no adjustment for multiple testing was considered. As the survey was most likely answered in a work setting, i.e., completion might have been interrupted (leading to longer answer times or early termination and restarting of the survey with shorter response times), time stamps were not analyzed.

### 2.6. Ethical Issues

The study adhered to the declaration of Helsinki. Approval by the local ethics committee of the medical faculty of Technical University of Munich (41/21 S), data protection officer, hospital board and staff counsel were obtained. Every participant gave informed consent by clicking a checkbox after the information on the study and data protection prior to the survey.

## 3. Results

### 3.1. Study Population

A total of 5448 participants started the online survey. Nine hundred and forty-eight surveys had to be excluded due to incompleteness or missing consent. Finally, the dataset consisted of 4500 completed surveys for analysis (Figure 1). The number of female participants was 2610 (58.0%). The largest participant groups were physicians with specialist/personnel responsibilities (29.5%), medical students (29.2%) and certified nurses (10.4%). The main work settings were maximum care hospitals/university hospitals (42.6%), hospitals of other care levels (18.0%) and medical practices/medical care centers (20.2%). Of all participants, 35.2% were from southern Germany, 28.1% were from northern Germany, 26.0% were from western Germany and 8.4% were from eastern Germany (Table 1).

### 3.2. Vaccination Acceptance in Different Age Groups/Work Settings and Regions

**Figure 2:**
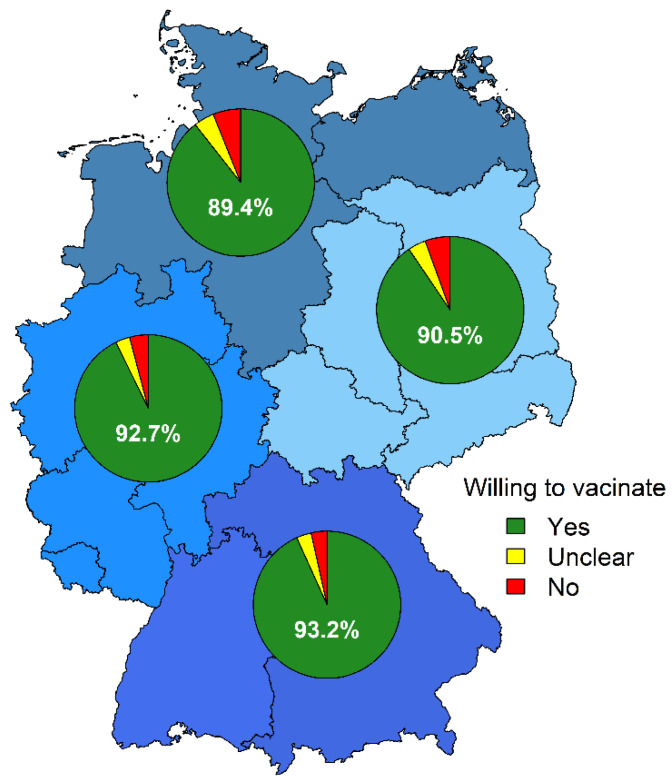
COVID-19 vaccination acceptance in German regions. Regions defined according to the region definitions in the COVIMO study [42]. Numbers of invitations, responses and inhabitants per region and federal country [43] can be found in Table S2.

**Figure 3:**
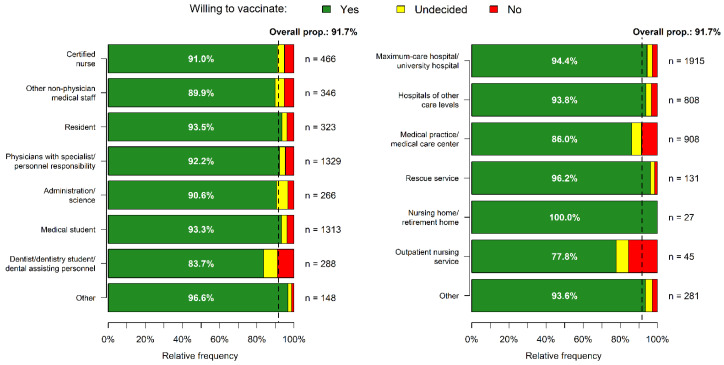
Vaccination acceptance in professional groups and work settings. The overall proportion of willing to be vaccinated was 91.7%. Abbreviations: prop., proportion.

**Figure 4:**
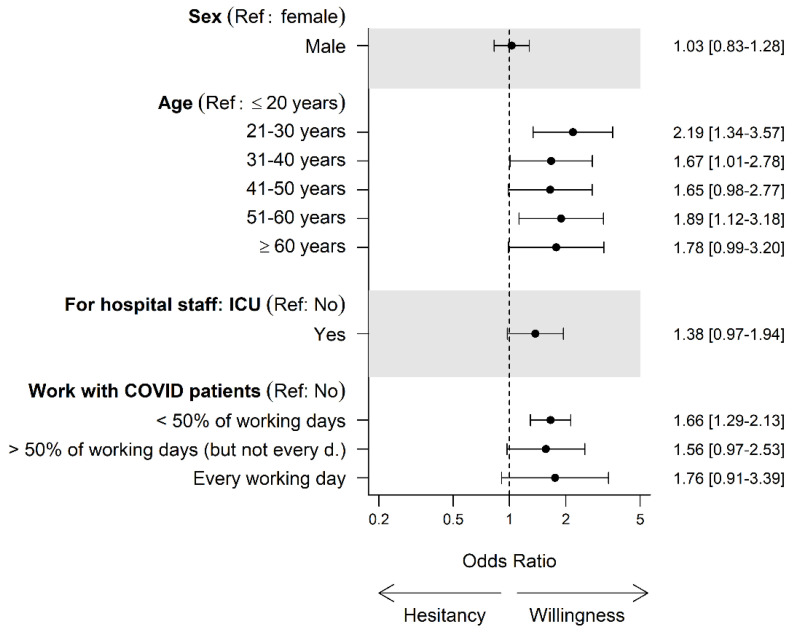
Basic demographics and vaccination acceptance. Abbreviations: Ref, reference; d., day; ICU, intensive care unit; COVID, coronavirus disease.

The overall vaccination acceptance was 91.7% (4125/4500): 167 (3.7%) participants were undecided, and 208 (4.6%) reported that they did not want to get vaccinated against COVID-19. Of those accepting a COVID-19 vaccination, 2024 (49.1%) had already received at least one COVID-19 vaccination dose. No difference in vaccination acceptance was observed between genders (Table 1). Among German regions, the vaccination acceptance ranged from 89.4% (northern Germany) to 93.2% (southern Germany, Figure 2). Participants from outpatient nursing services (10/45, 22.2% unwilling or undecided) and medical practices/medical care centers (127/908, 14.0%) were less likely to accept COVID-19 vaccination compared to participants from other work settings (Table 1, Figure 3). The age group ≤ 20 years showed the lowest vaccination acceptance. Among professional groups, dentistry personnel showed the lowest vaccination acceptance, with 47/288 (16.3%) being unwilling or undecided (Table 1, Figure 4).

### 3.3. Personal Attitudes and Vaccination Experience

Overall, most participants somewhat or fully agreed that they trust both vaccines in general (4199, 93.3%) and COVID-19 vaccines (3639, 80.9%). Most respondents somewhat or fully agreed that vaccines in general (4330, 96.2%) and COVID-19 vaccines (3961, 88.0%) are effective. Additionally, most of the respondents confidently or tentatively claimed to keep their vaccinations up to date (4150, 92.2%).

#### 3.3.1. Attitudes towards Authorities and Medical Institutions and COVID-19 Vaccination Hesitancy

**Figure 5:**
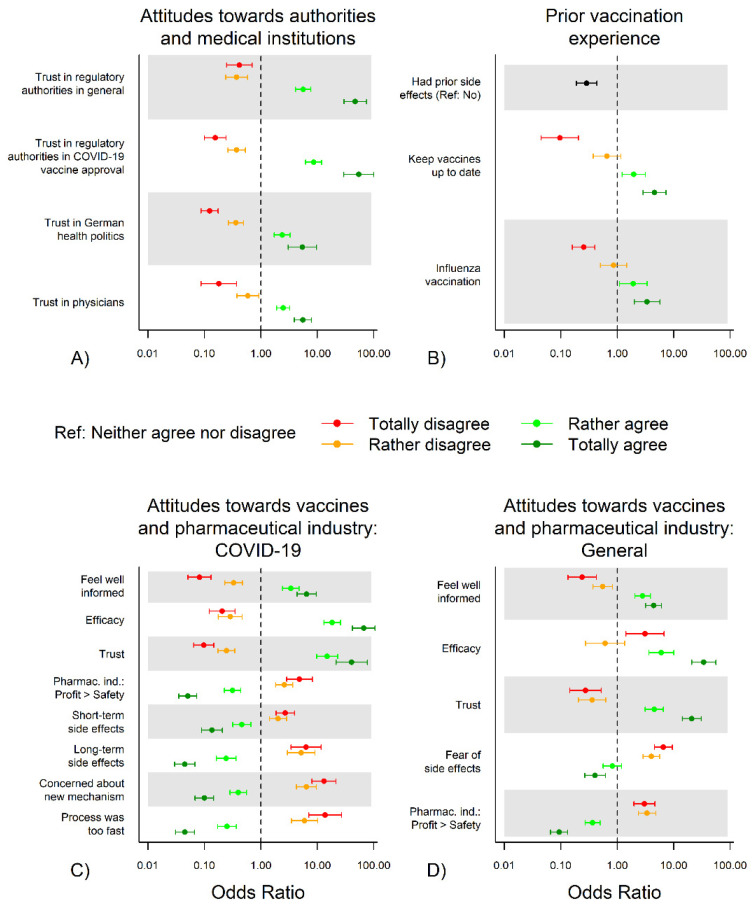
Attitudes toward and experiences with vaccinations. Attitudes towards authorities and medical institutions (**A**), prior vaccination experience (**B**), attitudes towards COVID-19-specific vaccines and the related parts of the pharmaceutical industry (**C**) and attitudes generally towards vaccines and the pharmaceutical industry (**D**). Abbreviations: Ref, reference; d., day; ICU, intensive care unit; incl., including; COVID-19, coronavirus disease 2019; Pharmac. ind., pharmaceutical industry. Numerical values of odds ratios with corresponding 95% confidence intervals are presented in Table S6.

Lack of trust in regulatory authorities in general was associated with vaccination hesitancy. In total, 115/198 (58.1%) of participants who totally or somewhat mistrusted authorities reported to be unwilling to get vaccinated or to be undecided; compared to 172/4041 (4.3%) of those who somewhat or totally trusted authorities (*p* < 0.001). Similarly, lack of trust in COVID-19 vaccine approval (181/322 (56.2%) vs. 85/3763 (2.3%), *p* < 0.001), in German health politics (206/762 (27.0%) vs. 85/2746 (3.1%), *p* < 0.001) and in physicians (51/164 (31.1%) vs. 224/3740 (6.0%), *p* < 0.001) were significantly associated with vaccine hesitancy. Odds ratios for comparison with the reference group of those who neither agreed nor disagreed are illustrated in Figure 5A; absolute and relative frequencies for all categories are presented in Table S5; odds ratios are presented in Table S6.

#### 3.3.2. Prior Vaccination Experience and COVID-19 Vaccination Hesitancy

A history of adverse vaccination effects (30/136 (22.1%) vs. 325/4323 (7.5%) of participants without prior adverse effects, *p* < 0.001), disagreement with generally keeping vaccines up to date (somewhat or totally disagreed: 74/201 (36.8%); somewhat or totally agreed: 269/4150 (6.5%), *p* < 0.001) and disagreement with regularly receiving vaccinations against influenza (275/1463 (18.8%) vs. 77/2713 (2.8%), *p* < 0.001) were associated with COVID-19 vaccine hesitancy (Figure 5B, Tables S5 and S6).

#### 3.3.3. Attitudes to (COVID-19) Vaccines and Pharmaceutical Industry and COVID-19 Vaccination Hesitancy

A strong association with COVID-19 vaccine hesitancy was found for fear of long-term (somewhat or totally agreed: 302/863 (35.0%); somewhat or totally disagreed: 35/3070 (1.1%), *p* < 0.001) and short-term (159/596 (26.7%) vs. 160/3377 (4.7%), *p* < 0.001) adverse effects of COVID-19 vaccines, and adverse vaccine effects in general (133/469 (28.4%) vs. 176/3705 (4.8%)). Similarly, disagreement on feeling well informed about vaccines in general (somewhat or totally disagreed: 85/265 (32.1%); somewhat or totally agreed: 219/3824 (5.7%), *p* < 0.001) and specifically on COVID-19 vaccines (174/398 (43.7%) vs. 135/3649 (3.7%), *p* < 0.001) was associated with vaccine hesitancy (Figure 5C, Tables S5 and S6).

Furthermore, increased vaccine hesitancy was found in participants who believed that financial profit is more important for the pharmaceutical industry than the safety of their products in general (somewhat or totally agreed: 197/663 (29.7%), somewhat or totally disagreed: 78/2713 (2.9%), *p* < 0.001) and the safety of COVID-19 vaccines (214/609 (35.1%) vs. 76/2854 (2.7%), *p* < 0.001). It was also associated with skepticism towards the velocity of COVID-19 vaccine development (299/695 (43.0%) vs. 31/3226 (1.0%), *p* < 0.001) or the new mechanism of action (237/584 (40.6%) vs. 61/3383 (1.8%), *p* < 0.001) (Figure 5C,D, Tables S5 and S6).

### 3.4. COVID-19-Specific Attitudes and Experiences in Personal Surroundings

**Figure 6:**
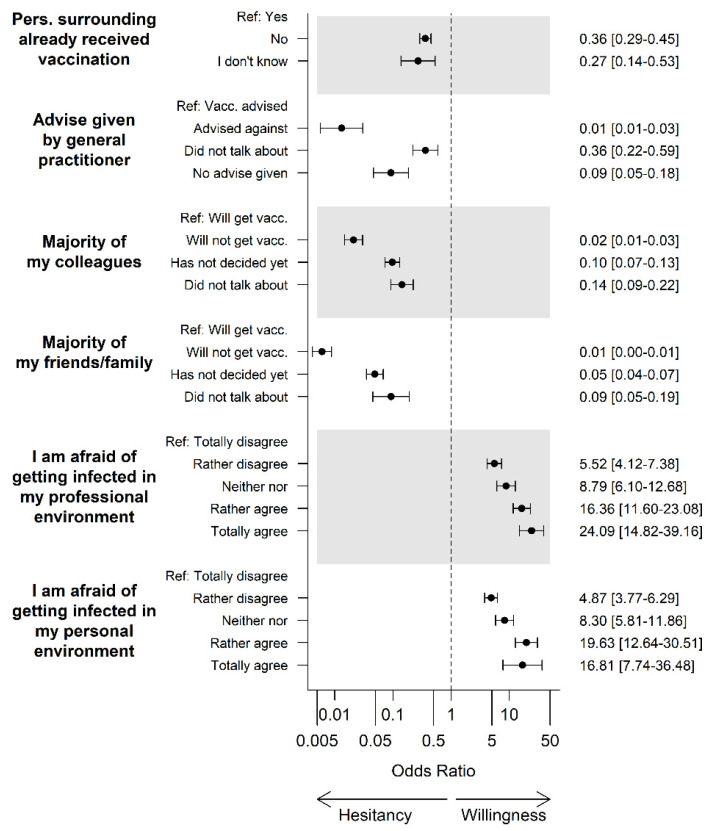
Personal environment and attitude towards COVID-19 vaccination. Abbreviations: Vacc., vaccination; GP, general practitioner; Pers., persons; Ref, reference.

A strong association with COVID-19 vaccine hesitancy was observed when participants stated that family and friends had decided not to get vaccinated (183/251, 72.9%). Only 59/3682 (1.6%) of those who reported that the majority of their families/friends have had or would like to have a COVID-19 vaccination were hesitant (*p* < 0.001). When their general practitioner had advised against COVID-19 vaccination, hesitancy was also more likely (30/41 (73.2%) vs. 18/518 (3.5%) with advice for vaccination, *p* < 0.001). Additionally, a strong association between participants’ colleagues’ decisions against COVID-19 vaccination and participants’ hesitancy was observed (participants with the majority of their colleagues rejecting COVID-19 vaccination: 106/173 (61.3%); participants with the majority of colleagues accepting COVID-19 vaccination: 116/3602 (3.2%), *p* < 0.001). If participants did not know whether persons in their surroundings had already been vaccinated against COVID-19 (11/56, 19.6%) or if none of them had been vaccinated (147/948, 15.5%), relevant vaccination hesitancy was present (217/3495 (6.2%) for participants who reported that people in their personal environments already received at least one vaccination dose; (both *p* < 0.001). Participants’ fear of infection in the professional environment (somewhat or totally agreed: 78/2307 (3.4%); somewhat or totally disagreed: 248/1451 (17.1%), *p* < 0.001) was more strongly associated with vaccine acceptance/hesitancy than their fear of infection in the private environment (32/1387 (2.3%) vs. 297/2225 (13.3%), *p* < 0.001) (Figure 6, Table S5).

**Figure 7:**
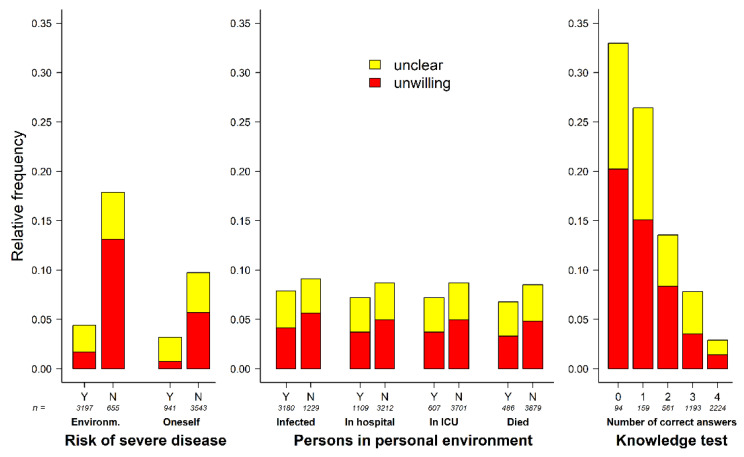
Perceived risk of severe COVID-19 disease, history of infections in personal environment and knowledge of COVID-19 vaccines. Abbreviations: ICU, intensive care unit; environm., environment; Y, yes; N, no. Risk of severe disease for oneself (totally agree to totally disagree) was dichotomized for visualization. Category “I do not know” was not visualized. All data are given in Table S5.

For participants expecting severe cases of COVID-19 in people they knew, the number of hesitant or undecided participants was 141/3197 (4.4%); compared to 117/655 (17.9%) of those not expecting it (*p* < 0.001; see Figure 7). Participants who felt that they were at risk of a severe case were significantly less often hesitant than participants not fearing a severe case of the disease for themselves (somewhat or totally agreed: 30/941 (3.2%); somewhat or totally disagreed: 292/2658 (11.0%), *p* < 0.001). Only small differences in vaccination acceptance/hesitancy were observed regarding history of COVID-19 infections, hospitalizations, intensive care unit admissions and deaths among the participants’ acquaintances. The number of correct answers in the knowledge test was significantly associated with the frequency of vaccine hesitancy (zero correct answers: 31/94, 33.0%; all four questions answered correctly: 64/2224, 2.9%, *p* < 0.001) (Figure 7, Table S5).

### 3.5. Main Sources of Information on COVID-19

**Figure 8:**
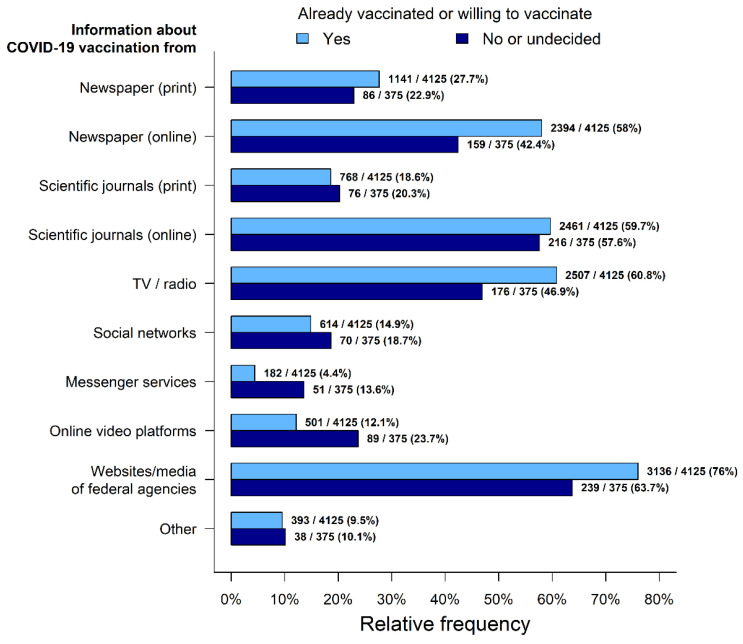
Source of information and media usage.

In the group of vaccine-hesitant participants, lower proportions reported obtaining COVID-19-vaccination-related information from online newspapers (42.4% vs. 58.0%, *p* < 0.001), TV/radio (46.9% vs. 60.8%, *p* < 0.001) or websites/media of federal agencies (63.7% vs. 76.0%, *p* < 0.001). Furthermore, in this group, it was more frequently reported that COVID-19-vaccination information was obtained via messenger services (13.6% vs. 4.4%, *p* < 0.001) or online video platforms (23.7% vs. 12.1%, *p* < 0.001) (Figure 8).

## 4. Discussion

### 4.1. Summary of Findings

We examined COVID-19 vaccine hesitancy in German healthcare workers (HCWs) in one of the largest and most comprehensive surveys performed. Overall, the data of 4500 German HCWs who participated in the survey during the second pandemic wave in Germany were considered for this analysis. Of these, about 92% were either willing to or already had received a COVID-19 vaccine. To summarize, participant groups of the lowest age, those who worked in outpatient nursing services or medical care centers/doctors’ practices and dentistry personnel were associated had the highest hesitancy rates. Furthermore, lack of trust in authorities, the vaccine approval process, the vaccines’ development velocity, health politics and the pharmaceutical industry were associated with hesitancy. Additionally, a history of vaccination side effects, not keeping vaccinations up to date, fear of long- and short-term side effects, lack of trust in the vaccines, not feeling well informed about vaccines in general and not feeling well informed about COVID-19 vaccines were associated with higher rates of hesitancy. Hesitancy in the personal surroundings (family, friends, colleagues, GP) was also associated with respondents’ hesitancy. Lastly, underperforming in the knowledge test and media usage types (online video platforms and messenger services) were associated with hesitancy. Interestingly, a history of acquaintances having suffered from COVID-19/having been hospitalized/admitted to ICU or even dying due to COVID-19 did not have a significant association with vaccine acceptance.

### 4.2. Overall Acceptance

General vaccine hesitancy in HCWs is common [8,44]. However, with 92% acceptance, we found broad consent to receive COVID-19 vaccination throughout all groups of HCWs. This stands in contrast to recently published studies in Germany and worldwide with acceptance rates between 31% and 86% [11,12,13,14,15,16,17,18,19,20,21,22,23,24,25,26,27] (see Supplementary Table S1) and shows that vaccine hesitancy and acceptance may change rapidly. The only comparable German survey was conducted mainly in intensive care settings. In that study, the acceptance of 50% by nurses and 73% by physicians was rather low compared to our data [16]. These low numbers would be insufficient to reach herd immunity in the medical sector [1]. However, the earlier assessment being in December 2020 with an absence of approved and available vaccines could explain these differences. One strength of this study is that the survey was conducted after the Comirnaty^®^ vaccine by BioNTech/Pfizer had been approved and was available for German HCWs in hospitals, and Vaxzevria was approved in Germany, though limited to certain age groups [45]. As vaccination campaigns had only started in January 2021 in most German hospitals, 44% of the participants in our study had already been vaccinated. As HCWs had the highest exposure and risk of disease transmission, they were ranked as high-priority and had a realistic chance of getting vaccinated very soon. The questions asked in other surveys were hypothetical, as no vaccines had been approved by healthcare authorities then. This might explain higher acceptance rates than in the literature. Furthermore, as our survey took place in February 2021, and therefore far later than the previously published studies, it is likely that the acceptance had changed; co-healthcare workers were already being vaccinated. HCWs often function as ambassadors for vaccine acceptance in the general population [44,46]. Therefore, our observations might lead to the conclusion that German HCWs might have already acted as role models for vaccine acceptance during the COVID-19 pandemic for their own peer groups. In addition, the peak of the second pandemic wave in Germany was just over [10], possibly leading to the strong wish to prevent a third wave.

### 4.3. Comparison of Vaccine Hesitancy in the German Healthcare Workers and the General Population

The COVID-19 vaccination acceptance in the general population in Germany is measured weekly by the COSMO study [47]. Acceptance rates have been relatively stable at 68% and therefore lower than in our survey [15]. The ongoing debate about Astra Zeneca’s vaccine Vaxzevria^®^ can only serve to a limited extent as an explanation for this discrepancy, since this debate (which developed due to several reasons) had just begun during our survey. Based on data available in February 2021, AstraZeneca’s COVID-19 vaccine was recommended in Germany for persons 18 to 64 years of age [45]. Furthermore, debates on the vaccine were due to rare cases of thrombocytopenia with cerebral venous sinus thrombosis and splanchnic vein thrombosis [48]. However, it seems conceivable that the threat from COVID-19 appears less abstract for HCWs than for the general population, which could lead to greater willingness to be vaccinated. However, preliminary results of the COVIMO study did not find major differences between German HCWs and the general population regarding acceptance of COVID-19 vaccination (preliminary results published online only [42]).

In Germany, influenza vaccinations are recommended to all HCWs [49]. In our survey, only 60% followed that recommendation, yet this rate was still higher than in some other published surveys of HCWs [49,50]. This suggests large-scale general vaccine acceptance in participating HCWs which is in line with the general trust in vaccines and their efficacy, and the high agreement with keeping vaccinations up to date. The perceived severe threat by COVID-19 might explain the higher acceptance of vaccination against COVID-19 as compared to the seasonal influenza.

### 4.4. Factors Associated with Vaccination Hesitancy

We could furthermore show that vaccine hesitancy in HCWs is multifactorial. However, comparisons with previously published studies, as shown in Table S1, need to be performed cautiously, as acceptance was defined differently, and measured on different scales, using different questionnaires with different numbers of items, in different countries and time periods [11,12,13,14,15,16,17,18,19,20,21,22,23,24,25,26,27].

As media reports on vaccine hesitancy in German HCWs claimed low acceptance rates, it became particularly important to assess the actual vaccination acceptance of HCWs and reasons for hesitancy. In our study, 3.7% of all participants were undecided and 4.6% openly refused COVID-19 vaccination.

In our study, the youngest age group showed the lowest vaccination acceptance. This is in line with the current literature, as Nohl et al. also stated that older age was associated with higher acceptance [25]. An explanation for this might be a lower risk of a severe case of the disease, but another could be the proclivity for risk-taking behavior in young people [51].

The subgroup of participants working in outpatient nursing services was more associated with COVID-19 vaccination hesitancy, with the caveat that there was a low number of participants in this subgroup. This could have been due to most vaccination campaigns taking place in hospitals and being driven by healthcare providers, the latter of which leads to the assumption that these HCWs were better informed.

Interestingly, both the group working in a medical practice or medical care center and dentistry personnel were more likely to refused a COVID-19 vaccination. This is surprising, considering that there is also a relevant risk of infection in the outpatient setting, especially in the dental field. One possible explanation may be that many physicians and dentists in private practice had treated only symptom-free patients, had significantly limited their treatment capacities and had introduced comprehensive hygiene concepts. If the 5C model of Betsch et al. [38] is taken into account, the effort toward receiving the vaccination could have played a role, in addition to the risk assessment. These groups had to take care of their own vaccine supplies, whereas many hospitals, especially maximum care hospitals, had their own vaccination centers.

Working with COVID-19 patients was not associated with acceptance of COVID-19 vaccination, except in HCWs with less than 50% occupation with COVID-19 patients. This might be explained by the perception of a higher transmission risk in the family setting than in the professional setting, where personal protective equipment and strict testing of patients and staff are used routinely, especially in COVID-19 treatment units, where HCWs treat COVID-19 patients daily. Additionally, it could be speculated that HCWs in German hospitals were sufficiently supplied with professional protective equipment, and therefore the threat of becoming infected with COVID-19 was judged as low [52].

It is not surprising that prior adverse effects to vaccines went along with lower vaccine acceptance. The same could be seen for the factor of “up to date” vaccination and the acceptance of the yearly influenza shot. This is in good accordance with previous studies [53]. General and COVID-19-vaccine-specific mistrust in the pharmaceutical industry, authorities and health politics were also associated with COVID-19 vaccine hesitancy. The distrust in the pharmaceutical industry, with regard to its financial interests, seems to have existed in European HCWs for some time [54]. Further studies are needed to examine both the reasons for the distrust in institutions and the frequency of its presence compared to the normal population, as distrust in institutions appears to be associated with lower vaccination acceptance and might therefore be harmful in the long term.

Distrust in the rapid development process has already been associated with lower vaccination acceptance in other studies as well [18,19,22].

Furthermore, fear of long- and short-term side effects and lack of trust in vaccines were associated with COVID-19 vaccination hesitancy. This is in line with most of the current literature [11,19,20,21,22,23,24,27].

Interestingly, hesitancy in personal surroundings (family, friends, colleagues, GP) was also associated with hesitancy in the respondents. Parental vaccination concerns are well-known [55]. However, attitudes toward vaccination in the personal environment seem to play a role not only in the parental sphere; even HCWs seem to factor this into their decisions. Whether improved information strategies among HCWs are sufficient to counteract this mistrust prevailing in the personal environment remains to be investigated.

The number of correct answers in the four COVID-19 vaccine knowledge questions was significantly associated with vaccination acceptance, providing a direct starting point for informational campaigns. This shows that not only the perceived level of information or knowledge [15,18] about vaccines, but also the actual level of knowledge is related to vaccination acceptance. Testing whether improving knowledge about COVID-19 vaccines is a modifiable risk factor leading to higher vaccination acceptance is recommended for further studies.

The type of media used (online video platforms and messenger services) was associated with hesitancy. The relationship between social media use and vaccine hesitancy had been shown already [56]. To counter this disinformation, more extensive information campaigns in the relevant channels are therefore likely to be required. The effectiveness of such measures should be studied in the future.

Lastly, a history of acquaintances having suffered from COVID-19/having been hospitalized/admitted to ICU or even dying due to COVID-19 did not have a significant association with vaccine acceptance. It seems possible that the experience of these complications did not have a strong influence on the factors collective responsibility and complacency mentioned by Betsch et al. [38]. However, it should be noted that it is not known whether the persons in the respondents’ circle of acquaintances had more comorbidities than the respondents themselves.

### 4.5. Possible Implications for COVID-19 Vaccination Campaigns

Overall, the acceptance in HCWs was remarkably high throughout Germany. There was a tendency toward less acceptance in northern and eastern parts of Germany. However, this small difference might not justify applying different vaccination campaigns in different German regions. Due to the high acceptance rate in HCWs, they should be involved as ambassadors for COVID-19 vaccination campaigns. We suppose that HCWs already serve as role models. Inclusion of HCWs in vaccination campaigns might contribute to improved trust in vaccinations. It seems that the low numbers of hesitant HCWs have high levels of distrust in politicians and health authorities. Having identified trust in vaccines and authorities as potential modifiable risk factors in HCWs, this might not be changeable in short-term campaigns, but will be important for long-term projects. Since the youngest group of respondents showed the lowest vaccination acceptance, this group should be targeted through information campaigns. As hesitant HCWs are using more video-streaming platforms and messenger services, it might be reasonable to use these media in a vaccination campaign. We assume that the content produced should be of high quality regarding content and storytelling. This implies the need for multi-disciplinary teams, including medical education media professionals. As knowledge about the COVID-19 vaccines is a strong factor in favor of vaccination, “infotainment” formats, e.g., easy to understand explanatory videos, might be useful. Importantly, to evaluate the effect of COVID-19 vaccination campaigns, scientific supervision appears to be advisable.

### 4.6. Limitations

Limitations of this study also need to be considered. Online surveys are a powerful tool to achieve broad responses in short periods of time, and therefore are especially of use in a pandemic situation [57]. This is opposed to conventional methods with pen and paper and careful selection of a representative sample, which would simply take too much time in extremely rapidly changing circumstances [57]. In addition, an online survey already seemed more than advisable given the distance requirements and the necessary reduction of interpersonal contact. However, as this was an open online survey, it is impossible to estimate how representative the sample is. As the major goal was to reach as many healthcare workers as possible via institutions and societies (snowball sampling [32]) and it could not be foreseen how the link would be shared and how many HCWs would be willing to participate, no formal sample size calculation was performed. However, we addressed as many HCWs as possible from every German region in order to build a strong dataset. Due to the broad distribution of the survey without addressing specific individuals (due to German data protection laws), we cannot estimate how many HCWs eventually received the invitation. Therefore, a response rate cannot be calculated. The number of invitations to the survey and the numbers of responses from German regions and federal states can be found in Supplementary Table S2. However, as several nationwide organizations were addressed, it is impossible to assess in which regions the invitation link was forwarded the most. The number of participants from Bavaria, 1380, was higher than in any other federal state, although the number of invitations to participate in the survey was not the highest, and Bavaria had only the second highest number of inhabitants. It can be assumed that the link was forwarded more often within Bavarian institutions and that Bavarian respondents felt more responsible to answer the survey as our university hospital is located in Munich, the capital of Bavaria. Selection bias is a common problem in online surveys [58], which cannot be ruled out. Since the study was based on an online survey, it could have been biased towards participants with a positive attitude towards a COVID-19 vaccination. In particular, mistrust in the state and organizations could further hinder participation. However, we kept the invitation text neutral so as not to discourage vaccine-critical individuals from participating. Furthermore, some vaccine-critical HCWs used this survey as an opportunity to express their opinions (we know this was at least sometimes true from some political statements in the comments). Although the survey was only sent to medical institutions, we cannot completely rule out that non-HCWs participated. However, given the heterogeneity in age structure, the occupational groups seeming to match the collective we contacted and the effort required to respond to this survey, we believe it is unlikely that the link was answered by a larger number of non-HCWs. Furthermore, IP address tracking would not prevent this residual risk.

In addition, it should be noted that due to the speed of developments during the pandemic, validation/reliability testing of our question set was impossible. However, we oriented our question set toward validated or already applied item sets [37,38,39,40]. Moreover, the results of individual items are comparable to the literature (apart from a higher overall vaccination rate). Furthermore, we have solely evaluated factors associated with vaccine hesitancy in HCWs. Therefore, implications may primarily be drawn for this target group and not be generalized to the general public. We recommend further research comparing these factors between HCWs and other population groups. Furthermore, due to the length of the survey, we decided not to add more knowledge test questions in order to avoid early termination of the survey by the respondents. We therefore chose questions that could be answered without special knowledge in order to check if failing to answer these questions is associated with vaccine hesitancy. We recommend further research on the importance of knowledge of COVID-19 vaccines in HCWs. Additionally, the small numbers in some subgroups allow only limited conclusions for these subgroups.

Last, our conclusions should be viewed in light of approval of the vaccines available during the survey by the regulatory authorities. Vaxzevria^®^ (not approved by the FDA at the time of the study) was approved in the European Union [59], and during the course of the study, by the World Health Organization [60]. Comirnaty^®^ was approved in both the USA [61] and Europe [62] during the study period. Our conclusions, especially the possible implications for vaccination campaigns, refer exclusively to vaccines that have been adequately tested and approved by the relevant regulatory authorities in the respective regions.

## 5. Conclusions

This study was one of the largest and most comprehensive studies on COVID-19 vaccination acceptance in HCWs of all working fields. In conclusion, we saw a high overall acceptance rate amongst HCWs for COVID-19 vaccination. Furthermore, several factors associated with vaccination hesitancy in HCWs were identified. These factors were: low age; working in outpatient nursing services or medical care centers/doctors’ practices; dentistry personnel; lack of trust in authorities, the vaccine approval process, the vaccine development velocity, health politics and the pharmaceutical industry; a history of vaccination side effects; not keeping vaccinations up to date; fear of long- and short-term side effects; lack of trust in the vaccines; not feeling well informed about vaccines in general and COVID-19 vaccines in particular; hesitancy in personal surroundings (family, friends, colleagues, GP); underperforming in the knowledge test; and the type of media usage (online video platforms and messenger services). These factors could be considered when designing new COVID-19 vaccination campaigns for HCWs. HCWs, due to their knowledge and overall high acceptance towards COVID-19 vaccination, may prove promising ambassadors for supporting COVID-19 vaccination campaigns.

## Figures and Tables

**Table 1:** Overall study population, and differences between participants undecided/dismissive or willing to receive COVID-19 vaccination.

Item	Total (n = 4500)	Undecided/Dismissive (n = 375)	Willing (n = 4125)	*p*
Sex				
Female	2610 (58.0)	218 (8.4)	2392 (91.6)	0.801
Male	1879 (41.8)	153 (8.1)	1726 (91.9)	
No answer	11 (0.2)			
Age				
≤20 years	156 (3.5)	22 (14.1)	134 (85.9)	0.039
21–30 years	1603 (35.6)	112 (7.0)	1491 (93.0)	
31–40 years	872 (19.4)	78 (8.9)	794 (91.1)	
41–50 years	730 (16.2)	66 (9.0)	664 (91.0)	
51–60 years	776 (17.2)	62 (8.0)	714 (92.0)	
≥61 years	355 (7.9)	30 (8.5)	325 (91.5)	
No answer	8 (0.2)			
Professional groups				
Certified nurse	466 (10.4)	42 (9.0)	424 (91.0)	<0.001
Other non-physician medical staff	346 (7.7)	35 (10.1)	311 (89.9)	
Resident	323 (7.2)	21 (6.5)	302 (93.5)	
Physicians with specialist/personnel responsibility	1329 (29.5)	104 (7.8)	1225 (92.2)	
Administration/science	266 (5.9)	25 (9.4)	241 (90.6)	
Medical student	1313 (29.2)	88 (6.7)	1225 (93.3)	
Dentist/dentistry student/dental assisting personnel	288 (6.4)	47 (16.3)	241 (83.7)	
Other	148 (3.3)	5 (3.4)	143 (96.6)	
No answer	21 (0.5)			
Work setting				
Maximum-care hospital/university hospital	1838 (40.8)	102 (5.5)	1736 (94.5)	<0.001
Hospitals of other care levels	792 (17.6)	47 (5.9)	745 (94.1)	
Medical practice/medical care center	771 (17.1)	107 (13.9)	664 (86.1)	
Rescue service	128 (2.8)	5 (3.9)	123 (96.1)	
Nursing home/retirement home	26 (0.6)	0 (0.0)	26 (100.0)	
Outpatient nursing service	34 (0.8)	10 (29.4)	24 (70.6)	
Other	526 (11.7)	47 (8.9)	479 (91.1)	
No answer	385 (8.6)			
Hospital, intensive care unit yes	1105 (24.6)	52 (4.7)	1053 (95.3)	0.070
Hospital, intensive care unit no	1525 (33.9)	97 (6.4)	1428 (93.6)	
Region				
Northern Germany	1266 (28.1)	134 (10.6)	1132 (89.4)	0.001
Southern Germany	1582 (35.2)	107 (6.8)	1475 (93.2)	
Eastern Germany	380 (8.2)	36 (9.5)	344 (90.5)	
Western Germany	1168 (26.0)	85 (7.3)	1083 (92.7)	
No answer	104 (2.3)			
COVID-19 patient care				
Never	1729 (38.4)	170 (9.8)	1559 (90.2)	<0.001
<50% of working days	1815 (40.3)	112 (6.2)	1703 (93.8)	
>50% of working days (but not every working day)	307 (6.8)	20 (6.5)	287 (93.5)	
On each working day	171 (3.8)	10 (5.8)	161 (94.2)	
No answer	478 (10.6)			

Results presented as numbers (percentage); *p*-values apply for overall group comparisons (Chi square test).

## Data Availability

The datasets for this manuscript are not publicly available because written informed consent excluded data sharing, as advised by the local data protection officer in accordance with the German data protection law.

## Supplementary Materials

The following are available online at https://www.mdpi.com/article/10.3390/vaccines9070777/s1. Table S1: Overview on studies identifying vaccination acceptance in medical professional. Table S2: Invitations and Responses from German regions and federal states. Table S3: Original questions of the survey, translated from German. Table S4: Application of the CHERRIES checklist [41]. Table S5: overall results. Table S6: Attitudes and experiences with vaccinations.
