# Supplementary material for: COVID-19 Vaccination Acceptance and Hesitancy among Healthcare Workers in Germany
Source: Vaccines (Basel). 2021 Jul 12;9(7):777. doi: 10.3390/vaccines9070777 (PMC8310090; doi:10.3390/vaccines9070777)
Supplement: Supplementary file 1 [file vaccines-09-00777-s001.zip › vaccines-1228317-supplementary.pdf]

Supplemental Material

Supplemental Table S1: Overview on studies identifying vaccination acceptance in medical professionals.

Publication	Country	Participants total	Subgroups	Acceptance (% of participants)	Hurdles / Barriers	Peer-reviewed	Survey conducted
Holzmann-Littig et al. (present manuscript)	Germany	4500	HCW	91.7%	development velocity, lack of trust in authorities, personal surrounding against vaccination, lack of information, generally not keeping vaccinations up to date	yes	Feb 21
Wang et al. [11]	Hong Kong	806	Nurses	40.3%	“suspicion on efficacy, effectiveness and safety”, “believing it unnecessary”, and “no time to take it”	yes	Feb / Mar 20
Kwok et al. [12]	Hong Kong	1205	Nurses	63.0%	5 C	yes	Mar / Apr 20
Dror et al. [13]	Israel	211	Nurses	61.1%	No care of COVID-19 patients	yes	Mar / Apr 20
Gagneux-Brunon [14]	France	1554	HCW	76.9%	vaccine hesitancy	yes	Mar-Jul 20
COSMO [9, 15]	Germany	127	n.a.	~64 %	Knowledge / information, trust	no	Dec 20
Karagiannidis et al. [16]	Germany	2305	Physicians, Nurses	64%		yes	Dec 20
DIVI/DGIIN [17]	Germany	3501	Physicians, Nurses	75%		no	Feb 21
American Nurses Foundation [18]	USA	12939	Nurses	34.0%	Development to quick, not enough information, unclear clinical trials process	no	Oct 20
Revive Health [19]	USA	370	Physicians, Nurses	31.0%	Safety, efficacy, compromised development process	no	Oct / Nov 20
Di Gennaro et al [20]	Italy	1723	HCW	67%	Lack of trust in vaccine safety, little / conflicting information	yes	Oct/Nov 20
Verger et al. [21]	France, Belgium, Quebec (Canada)	2678	HCW	71.6%	Vaccine safety concerns	yes	Oct / Nov 20
Papagiannis et al. [22]	Greece	340	HCW	78.5%	fear of adverse effects, shortness of vaccine development time	yes	Dec 20
Meyer et al. [23]	Pennsylvania, USA	16158	HCW	55.0%	Unknown risks, insufficient data, adverse effects, wanting to wait to see how it goes with others	Preprint	Dec 20
Dzieciolowska et al. [26]	Canada	2761	HCW	80.9%	Vaccine novelty, wanting others to receive it first, insufficient time for decision making	yes	Dec 20
Nohl et al. [25]	Germany	1296	EMS staff	57.0%	Female gender, lower education level, younger age, less firsthand experience with disease burden	yes	Dec 20-Jan 21
Schrading et al. [24]	USA	1398	HCW	86.0%	Safety concerns	yes	Jan 21
Aloweidi et al. [27]	Jordan	646	HCW / non-HCW	45.6 (HCW)	Safety concerns, trust	yes	Jan 21-Feb 21

Abbreviations: GP, general practitioner; DIVI, Deutsche Interdisziplinäre Vereinigung für Intensiv- und Notfallmedizin; DGIIN, Deutsche Gesellschaft [illegible] Internistische Intensivmedizin und Notfallmedizin; HCW, health care worker; n.a., not applicable, EMS: emergency medical services. The search strate[illegible] included these main components: 'vaccine hesitancy', 'health care workers' and "Covid-19". "Health care workers" was substituted with "health ca[illegible] professionals" in another search. We searched: Pubmed, medRxiv.org for preprints, Web of Science and Google Scholar to find relevant studies published [illegible] to March 2021. We included studies published in the English and German language. Google Search was used to identify press releases of ongoing a[illegible] unpublished studies. These were only included if they were either published in a peer-reviewed journal or preprint. From the identified publications and preprin[illegible] we checked wether studies on the same topic that were cited in these publications had been included in our overview of studies already. The table is intend[illegible] to provide an overview without claiming to be fully comprehensive. Additional studies have been added in May 2021.

Supplemental Table S2: Invitations and responses from German regions and federal states

Federal state	Number of e-mail invitations (% of total)	Number of responses in the final dataset (% of total)	Number of inhabitants Dec. 31st, 2019 [Millions], (% of total)
**Northern Germany**			
Schleswig-Holstein	183 (4.7)	516 (11.5)	2.904 (3.5)
Mecklenburg-Western Pomerania	174 (4.4)	34 (0.8)	1.608 (1.9)
Hamburg	171 (4.3)	94 (2.1)	1.847 (2.2)
Lower Saxony	227 (5.8)	593 (13.2)	7.994 (9.6)
Bremen	152 (3.9)	29 (0.6)	0.681 (0.8)
Total Northern Germany	907 (23.1)	1266 (28.1)	15.034 (18.1)
**Eastern Germany**			
Brandenburg	195 (5.0)	81 (1.8)	2.522 (3.0)
Berlin	196 (5.0)	110 (2.4)	3.669 (4.4)
Saxony-Anhalt	174 (4.4)	61(1.4)	2.195 (2.6)
Saxony	212 (5.4)	81 (1.8)	4.072 (4.9)
Thuringia	167 (4.3)	47 (1.0)	2.133 (2.6)
Total Eastern Germany	944 (24.1)	380 (8.4)	14.591 (17.5)
**Western Germany**			
North Rhine-Westphalia	292(7.4)	643 (14.3)	17.947 (21.6)
Hesse	229 (5.8)	400 (8.9)	6.288 (7.6)
Rhineland-Palatinate	223 (5.7)	94 (2.1)	4.094 (4.9)
Saarland	150 (3.8)	31 (0.7)	0.987 (1.2)
Total Western Germany	894 (22.8)	1168 (26.0)	29.316 (35.2)
**Southern Germany**			
Bavaria	375 (9.6)	1380 (30.7)	13.125 (15.8)
Baden-Wuerttemberg	704 (17.9)	202 (4.5)	11.100 (13.3)
Total Southern Germany	1079 (27.5)	1582 (35.2)	24.225 (29.1)
**Nationwide**			
Non region related organizations:	100		
Germany total	3924		83.166
No answer to federal state question		104 (2.3)	
Datasets used		4500	

Supplemental Table S3. Original questions of the survey, translated from German.

**Section 1: Basic data / demographics**
**Sex**
female / male / no answer
**Age**
≤20 years / 21-30 years / 31 - 40 years / 41 - 50 years / 51 - 60 years / ≥61 years / no answer
**Profession**
Certified nurse / Non-examined nurse / Medical specialist (Medical technical assistants, surgical assistant etc.) / Resident physician / specialized physician / Consultant physician / Chief physician / administrative staff / Non-physician staff in the rescue service / Trainee / student of human medicine / Student of dentistry / other – Free text / no answer
**Work Setting**
Maximum-care hospital; university hospital / Hospitals of other care levels<br>Medical practice; Medical care center / Rescue service / nursing home; retirement home / Outpatient nursing service / other – free text / no answer
**You indicated that you work in an inpatient setting. Do you work in an intensive care unit in this setting? If work setting 1 or 2 have been selected.**
yes / no
**I work with COVID-19 positive patients**
Never / under 50% of my working days / over 50% of my working days (but not every working day) / on each of my working days / no answer
**I work in the following federal district:**
Bavaria / Baden-Wuerttemberg / Berlin / Brandenburg / Bremen / Hamburg / Hesse / Mecklenburg-Western Pomerania / Lower Saxony / North Rhine-Westphalia / Rhineland-Palatinate / Saarland / Saxony / Saxony-Anhalt / Schleswig-Holstein / Thuringia / no answer
**Section 2: Vaccinations in general**
**Please rate the following statements about vaccinations in general:**
**I make sure to keep my vaccinations up to date.**
I do not agree at all / I rather do not agree / I neither agree nor disagree / I rather agree / I fully agree / no answer
**I receive flu (influenza) vaccinations regularly.**
I do not agree at all / I rather do not agree / I neither agree nor disagree / I rather agree / I fully agree / no answer
**I feel well informed about vaccines in general.**
I do not agree at all / I rather do not agree / I neither agree nor disagree / I rather agree / I fully agree / no answer
**I am generally afraid of adverse effects of vaccinations.**
I do not agree at all / I rather do not agree / I neither agree nor disagree / I rather agree / I fully agree / no answer
**I think vaccinations are generally effective.**
I do not agree at all / I rather do not agree / I neither agree nor disagree / I rather agree / I fully agree / no answer
**I trust vaccines in general.**
I do not agree at all / I rather do not agree / I neither agree nor disagree / I rather agree / I fully agree / no answer
**I believe that the pharmaceutical industry puts profit motives over the safety of vaccines.**
I do not agree at all / I rather do not agree / I neither agree nor disagree / I rather agree / I fully agree / no answer
**I trust the regulatory authorities of vaccines in Germany in general.**
I do not agree at all / I rather do not agree / I neither agree nor disagree / I rather agree / I fully agree / no answer
**I already had a serious vaccine adverse effect that required medical treatment.**
yes / no / no answer, if yes -> free text

Section 3 Vaccinations against COVID-19

I have already received one or two vaccinations against COVID-19 or already have a vaccination appointment.

yes / no / no answer

In my personal environment (family, friends, colleagues) there are people who have already received one (or two) vaccinations.

yes / no / I do not know / no answer

I want to get vaccinated against COVID-19. (Questions opens only if participant indicates not to have been vaccinated yet / if participant clicked "no answer".)

yes / no / undecided / no answer

I do not want to get vaccinated until I see that a lot of people who have been vaccinated have tolerated it. (Question only opens, if "undecided" or "no answer" have been indicated.

I do not agree at all / I rather do not agree / I neither agree nor disagree / I rather agree / I fully agree / no answer

In my personal environment (family, friends, colleagues) there were people suffering from COVID-19.

yes / no / I do not know / no answer

You have indicated that there were COVID-19 patients in your personal environment. Please answer the following questions: (Question only opens, if "yes" has been indicated.)
One / more persons of the above have been hospitalized in connection with COVID-19 disease

yes / no / I do not know / no answer

One / more persons of the above have been in an intensive care unit in connection with the COVID-19 disease

yes / no / I do not know / no answer

One / more persons of the above have died in connection with COVID-19 disease

yes / no / I do not know / no answer

In my personal environment (family, friends, colleagues) there are people for whom a COVID-19 disease would probably be severe.

yes / no / I do not know / no answer

Please answer the following questions on COVID-19 / vaccinations against COVID-19:

In case of COVID-19 disease, I fear a severe course of the disease for myself

I do not agree at all / I rather do not agree / I neither agree nor disagree / I rather agree / I fully agree / no answer

I am afraid of getting infected with COVID-19 in my professional environment.

I do not agree at all / I rather do not agree / I neither agree nor disagree / I rather agree / I fully agree / no answer

I am afraid of becoming infected with COVID-19 in a private environment.

I do not agree at all / I rather do not agree / I neither agree nor disagree / I rather agree / I fully agree / no answer

I feel well informed about COVID-19 vaccines.

I do not agree at all / I rather do not agree / I neither agree nor disagree / I rather agree / I fully agree / no answer

I am afraid of short-term adverse effects of COVID-19 vaccines.

I do not agree at all / I rather do not agree / I neither agree nor disagree / I rather agree / I fully agree / no answer

I am afraid of long-term adverse effects from COVID-19 vaccines.

I do not agree at all / I rather do not agree / I neither agree nor disagree / I rather agree / I fully agree / no answer

I believe the COVID-19 vaccines are effective.

I do not agree at all / I rather do not agree / I neither agree nor disagree / I rather agree / I fully agree / no answer

I am concerned about what I consider novel mechanisms of action in COVID-19 vaccines.

I do not agree at all / I rather do not agree / I neither agree nor disagree / I rather agree / I fully agree / no answer

The development and approval of COVID-19 vaccines are moving too fast for me.

I do not agree at all / I rather do not agree / I neither agree nor disagree / I rather agree / I fully agree / no answer

I generally trust the COVID-19 vaccines.

I do not agree at all / I rather do not agree / I neither agree nor disagree / I rather agree / I fully agree / no answer

I believe the pharmaceutical industry cares more about its profit motives than safety of COVID-19 vaccines.

I do not agree at all / I rather do not agree / I neither agree nor disagree / I rather agree / I fully agree / no answer

I trust the European regulatory authorities of COVID-19 vaccines used in Germany.

I do not agree at all / I rather do not agree / I neither agree nor disagree / I rather agree / I fully agree / no answer

I generally trust in the German health care politics.

I do not agree at all / I rather do not agree / I neither agree nor disagree / I rather agree / I fully agree / no answer

I generally trust in the medical opinion of physicians.

I do not agree at all / I rather do not agree / I neither agree nor disagree / I rather agree / I fully agree / no answer

I favor an introduction of compulsory vaccination for health care professionals.

I do not agree at all / I rather do not agree / I neither agree nor disagree / I rather agree / I fully agree / no answer

In case of approval by the European and German authorities, I would also be vaccinated with a vaccine not developed in America or Europe.

I do not agree at all / I rather do not agree / I neither agree nor disagree / I rather agree / I fully agree / no answer

If you are concerned about short-term adverse effects, what are they?

Free text

If you are concerned about long-term adverse effects, what are they?

Free text

Knowledge

Which of the statements about COVID-19 vaccines do you think are correct?

m-RNA vaccines stimulate the formation of protein structures that mimic parts of the viral envelope.

true / false / I do not know / no answer

m-RNA vaccines alter the genetic material

true / false / I do not know / no answer

COVID-19 vaccination can cause me to shed infectious viruses

true / false / I do not know / no answer

m-RNA is rapidly degraded in the body

true / false / I do not know / no answer

My primary care physician has given me ...

advise to get vaccinated against COVID-19 / advise not to get vaccinated COVID-19 / We have not discussed the vaccination so far. / We have addressed the vaccination; my primary care physician has not made a recommendation for or against the vaccination. / no answer

The majority of my colleagues have / would like to

get vaccinated against COVID-19 / not get vaccinated against COVID-19 / not yet decided whether to get vaccinated / we have not discussed vaccination so far / answer

The majority of my family / friends have or would like to

get vaccinated against COVID-19 / not get vaccinated against COVID-19 / not yet decided whether to get vaccinated / we have not discussed vaccination so far / no answer

I have given the majority of my patients (without contraindication) ...

advise to get vaccinated against COVID-19 / advise not to get vaccinated COVID-19 / We have not discussed the vaccination so far / We have addressed the vaccination; I have not made a recommendation for or against the vaccination / no answer

I have given my family / friends

advise to get vaccinated against COVID-19 / advise not to get vaccinated COVID-19 / We have not discussed the vaccination so far / We have addressed the vaccination; I have not made a recommendation for or against the vaccination / no answer

Because of the COVID-19 pandemic, I, or my family, have suffered economic losses.

yes / no / I do not know / no answer

From what media do you get your information on COVID-19 vaccination?

Daily newspapers, weekly magazines (print) / Daily newspapers, weekly magazines (online) / Scientific journals (print) / Scientific journals (online) / Television / radio / social networks (e.g., Facebook) / messenger services (e.g., Twitter, Telegram) / Online video platforms (e.g., YouTube) / Websites / information portals of government health authorities (e.g., RKI, WHO) / Other -> Free text

Supplemental Table S4: Appliance of the CHERRIES checklist, table adapted from the Cherries checklist (29)

Item Category	Reported in materials / methods
**Design**	
Describe survey design	√
**IRB (Institutional Review Board) approval and informed consent process**	
IRB approval	√
Informed consent	√
Data protection	√
**Development and pre-testing**	
Development and testing	√
**Recruitment process and description of the sample having access to the questionnaire**	
Open survey versus closed survey	√
Contact mode	√
Advertising the survey	√
**Survey administration**	
Web/E-mail	√
Context	√
Mandatory/voluntary	√
Incentives	√
Time/Date	√
Randomization of items or questionnaires	√
Adaptive questioning	√
Number of Items	√
Number of screens (pages)	√
Completeness check	√
Review step	√
**Response rates**	
Unique site visitor	n/a
View rate (Ratio of unique survey visitors/unique site visitors)	n/a
Participation rate (Ratio of unique visitors who agreed to participate/unique first	n/a
Completion rate (Ratio of users who finished the survey/users who agreed to	√
**Preventing multiple entries from the same individual**	
Cookies used	√
IP check	√
Log file analysis	√

Item Category	Reported in materials / methods
Registration	√
**Analysis**	
Handling of incomplete questionnaires	√
Questionnaires submitted with an atypical timestamp	√
Statistical correction	√

Supplemental Table S5: overall results.

Sex	Want to get vaccinated or already vaccinated (N=4125)	%	Undecided (N=167)	%	Do not want to get vaccinated (N=208)	%	Total (N=4500)	%
Female	2392	91.6	115	4.4	103	3.9	2610	100.0
Male	1726	91.9	51	2.7	102	5.4	1879	100.0
Missing / no answer							11	
**Age**								
≤20 years	134	85.9	8	5.1	14	9.0	156	100.0
21-30 years	1491	93.0	51	3.2	61	3.8	1603	100.0
31-40 years	794	91.1	39	4.5	39	4.5	872	100.0
41-50 years	664	91.0	30	4.1	36	4.9	730	100.0
51-60 years	714	92.0	29	3.7	33	4.3	776	100.0
≥61 years	325	91.5	8	2.3	22	6.2	355	100.0
Missing / no answer							8	
**Profession**								
Certified nurse	424	91.0	18	3.9	24	5.2	466	100.0
Other non-physician medical staff	311	89.9	17	4.9	18	5.2	346	100.0
Resident	302	93.5	9	2.8	12	3.7	323	100.0
Physician with specialist / personnel responsibility	1225	92.2	42	3.2	62	4.7	1329	100.0
Administration / science	241	90.6	16	6.0	9	3.4	266	100.0
Medical student	1225	93.3	37	2.8	51	3.9	1313	100.0
Dentist / dentistry student / dental assisting personnel	241	83.7	21	7.3	26	9.0	288	100.0
Other non-physician medical staff	143	96.6	3	2.0	2	1.4	148	100.0
Missing / no answer							21	
**Work setting**								
Maximum-care hospital / university hospital	1807	94.4	55	2.9	53	2.8	1915	100.0
Hospital of other care levels	758	93.8	23	2.8	27	3.3	808	100.0
Medical practice / medical care center	781	86.0	49	5.4	78	8.6	908	100.0
Rescue service	126	96.2	3	2.3	2	1.5	131	100.0
Nursing home / retirement home	27	100.0	0	0.0	0	0.0	27	100.0
Outpatient nursing service	35	77.8	3	6.7	7	15.6	45	100.0
Other non-physician medical staff	263	93.6	10	3.6	8	2.8	281	100.0

Missing / no answer							385	
**You indicated that you work in an inpatient setting. Do you work in an intensive care unit in this setting?**								
Yes	1053	95.3	20	1.8	32	2.9	1105	100.0
No	1428	93.6	55	3.6	42	2.8	1525	100.0
Missing / no answer							1870	
**I work with COVID-19 positive patients**								
Never	1559	90.2	80	4.6	90	5.2	1729	100.0
< 50% of working days	1703	93.8	46	2.5	66	3.6	1815	100.0
> 50% of working days (but not every working day)	287	93.5	7	2.3	13	4.2	307	100.0
On each working day	161	94.2	2	1.2	8	4.7	171	100.0
Missing / no answer							478	
**Region**								
Southern Germany	1475	93.2	52	3.3	55	3.5	1582	100.0
Eastern Germany	344	90.5	15	3.9	21	5.5	380	100.0
Northern Germany	1132	89.4	57	4.5	77	6.1	1266	100.0
Western Germany	1083	92.7	37	3.2	48	4.1	1168	100.0
Missing / no answer							104	
**I make sure to keep my vaccinations up to date.**								
Totally disagree	14	29.2	4	8.3	30	62.5	48	100.0
Rather disagree	113	73.9	8	5.2	32	20.9	153	100.0
Neither agree nor disagree	107	81.1	11	8.3	14	10.6	132	100.0
Rather agree	1043	89.4	69	5.9	55	4.7	1167	100.0
Totally agree	2838	95.1	73	2.4	72	2.4	2983	100.0
Missing / no answer							17	
**I receive flu (influenza) vaccinations regularly**								
Totally disagree	722	75.8	85	8.9	146	15.3	953	100.0
Rather disagree	466	91.4	31	6.1	13	2.5	510	100.0
Neither agree nor disagree	271	92.5	9	3.1	13	4.4	293	100.0
Rather agree	758	95.9	19	2.4	13	1.6	790	100.0
Totally agree	1878	97.7	23	1.2	22	1.1	1923	100.0
Missing / no answer							31	
**I feel well informed about vaccines in general.**								
Totally disagree	30	52.6	8	14.0	19	33.3	57	100.0
Rather disagree	150	72.1	24	11.5	34	16.3	208	100.0
Neither agree nor disagree	331	82.3	43	10.7	28	7.0	402	100.0
Rather agree	1576	92.9	64	3.8	56	3.3	1696	100.0
Totally agree	2029	95.3	28	1.3	71	3.3	2128	100.0
Missing / no answer							9	
**I am generally afraid of adverse effects of vaccinations.**								
Totally disagree	1851	96.3	28	1.5	43	2.2	1922	100.0
Rather disagree	1678	94.1	56	3.1	49	2.7	1783	100.0

Neither agree nor disagree	254	79.9	29	9.1	35	11.0	318	100.0
Rather agree	241	76.5	39	12.4	35	11.1	315	100.0
Totally agree	95	61.7	15	9.7	44	28.6	154	100.0
Missing / no answer							8	
**I think vaccinations are generally effective.**								
Totally disagree	30	66.7	1	2.2	14	31.1	45	100.0
Rather disagree	13	28.3	5	10.9	28	60.9	46	100.0
Neither agree nor disagree	29	39.2	8	10.8	37	50.0	74	100.0
Rather agree	456	79.6	66	11.5	51	8.9	573	100.0
Totally agree	3594	95.7	87	2.3	76	2.0	3757	100.0
Missing / no answer							5	
**I trust vaccines in general.**								
Totally disagree	18	31.0	0	0.0	40	69.0	58	100.0
Rather disagree	30	37.0	15	18.5	36	44.4	81	100.0
Neither agree nor disagree	97	62.2	27	17.3	32	20.5	156	100.0
Rather agree	1038	88.2	80	6.8	59	5.0	1177	100.0
Totally agree	2937	97.2	45	1.5	40	1.3	3022	100.0
Missing / no answer							6	
**I believe that the pharmaceutical industry puts profit motives over safety of vaccines.**								
Totally disagree	914	96.9	12	1.3	17	1.8	943	100.0
Rather disagree	1721	97.2	33	1.9	16	0.9	1770	100.0
Neither agree nor disagree	976	91.2	53	5.0	41	3.8	1070	100.0
Rather agree	369	79.2	44	9.4	53	11.4	466	100.0
Totally agree	97	49.2	21	10.7	79	40.1	197	100.0
Missing / no answer							54	
**I trust the regulatory authorities of vaccines in Germany in general.**								
Totally disagree	34	43.6	5	6.4	39	50.0	78	100.0
Rather disagree	49	40.8	12	10.0	59	49.2	120	100.0
Neither agree nor disagree	161	64.9	41	16.5	46	18.5	248	100.0
Rather agree	1513	91.3	94	5.7	51	3.1	1658	100.0
Totally agree	2356	98.9	15	0.6	12	0.5	2383	100.0
Missing / no answer							13	
**I already had a serious vaccine adverse effect that required medical treatment.**								
Yes	106	77.9	11	8.1	19	14.0	136	100.0
No	3998	92.5	151	3.5	174	4.0	4323	100.0
Missing / no answer							41	
**In my personal environment (family, friends, colleagues) there are people who have already received one (or two) vaccinations.**								
Yes	3278	93.8	104	3.0	113	3.2	3495	100.0
No	801	84.5	58	6.1	89	9.4	948	100.0
I do not know	45	80.4	5	8.9	6	10.7	56	100.0
Missing / no answer							1	
**In my personal environment (family, friends, colleagues) there were people suffering from COVID-19.**								
Yes	2929	92.1	119	3.7	132	4.2	3180	100.0
No	1117	90.9	43	3.5	69	5.6	1229	100.0
I do not know	77	86.5	5	5.6	7	7.9	89	100.0
Missing / no answer							2	

**One / more persons of the above have been hospitalized in connection with COVID-19 disease**								
Yes	1029	92.8	39	3.5	41	3.7	1109	100.0
No	2933	91.3	120	3.7	159	5.0	3212	100.0
I do not know	78	95.1	3	3.7	1	1.2	82	100.0
Missing / no answer							97	
**One / more persons of the above have been in an intensive care unit in connection with the COVID-19 disease**								
Yes	566	93.2	21	3.5	20	3.3	607	100.0
No	3386	91.5	136	3.7	179	4.8	3701	100.0
I do not know	88	93.6	4	4.3	2	2.1	94	100.0
Missing / no answer							98	
**One / more persons of the above have died in connection with COVID-19 disease**								
Yes	456	93.8	14	2.9	16	3.3	486	100.0
No	3550	91.5	145	3.7	184	4.7	3879	100.0
I do not know	32	97.0	0	0.0	1	3.0	33	100.0
Missing / no answer							102	
**In my personal environment (family, friends, colleagues) there are people for whom a COVID-19 disease would probably be severe**								
Yes	3056	95.6	87	2.7	54	1.7	3197	100.0
No	538	82.1	31	4.7	86	13.1	655	100.0
I do not know	515	82.1	49	7.8	63	10.0	627	100.0
Missing / no answer							21	
**In case of COVID-19 disease, I fear a severe course of the disease for myself**								
Totally disagree	444	74.4	30	5.0	123	20.6	597	100.0
Rather disagree	1922	93.3	86	4.2	53	2.6	2061	100.0
Neither agree nor disagree	832	94.0	28	3.2	25	2.8	885	100.0
Rather agree	651	96.9	17	2.5	4	0.6	672	100.0
Totally agree	260	96.7	6	2.2	3	1.1	269	100.0
Missing / no answer							16	
**I am afraid of getting infected with COVID-19 in my professional environment.**								
Totally disagree	203	60.8	23	6.9	108	32.3	334	100.0
Rather disagree	1000	89.5	62	5.6	55	4.9	1117	100.0
Neither agree nor disagree	654	93.2	27	3.8	21	3.0	702	100.0
Rather agree	1445	96.2	41	2.7	16	1.1	1502	100.0
Totally agree	784	97.4	14	1.7	7	0.9	805	100.0
Missing / no answer							40	
**I am afraid of becoming infected with COVID-19 in a private environment.**								
Totally disagree	334	69.0	27	5.6	123	25.4	484	100.0
Rather disagree	1594	91.6	88	5.1	59	3.4	1741	100.0
Neither agree nor disagree	832	94.9	29	3.3	16	1.8	877	100.0
Rather agree	1093	97.8	19	1.7	6	0.5	1118	100.0
Totally agree	262	97.4	4	1.5	3	1.1	269	100.0
N-Miss							11	
**I feel well informed about COVID-19 vaccines.**								
Totally disagree	37	32.5	14	12.3	63	55.3	114	100.0

Rather disagree	187	65.8	61	21.5	36	12.7	284	100.0
Neither agree nor disagree	380	85.4	36	8.1	29	6.5	445	100.0
Rather agree	1770	95.2	43	2.3	46	2.5	1859	100.0
Totally agree	1744	97.4	13	0.7	33	1.8	1790	100.0
Missing / no answer							8	
**I am afraid of short-term adverse effects of COVID-19 vaccines.**								
Totally disagree	1558	95.9	29	1.8	37	2.3	1624	100.0
Rather disagree	1659	94.6	51	2.9	43	2.5	1753	100.0
Neither agree nor disagree	461	89.7	23	4.5	30	5.8	514	100.0
Rather agree	353	80.0	48	10.9	40	9.1	441	100.0
Totally agree	84	54.2	16	10.3	55	35.5	155	100.0
Missing / no answer							13	
**I am afraid of long-term adverse effects of COVID-19 vaccines.**								
Totally disagree	1451	99.0	4	0.3	11	0.8	1466	100.0
Rather disagree	1584	98.8	16	1.0	4	0.2	1604	100.0
Neither agree nor disagree	519	93.9	18	3.3	16	2.9	553	100.0
Rather agree	434	78.8	66	12.0	51	9.3	551	100.0
Totally agree	127	40.7	62	19.9	123	39.4	312	100.0
Missing / no answer							14	
**I believe the COVID-19 vaccines are effective.**								
Totally disagree	22	22.2	4	4.0	73	73.7	99	100.0
Rather disagree	29	28.4	16	15.7	57	55.9	102	100.0
Neither agree nor disagree	180	58.1	82	26.5	48	15.5	310	100.0
Rather agree	1656	96.2	47	2.7	18	1.0	1721	100.0
Totally agree	2216	98.9	16	0.7	8	0.4	2240	100.0
Missing / no answer							28	
**I am concerned about what I consider novel mechanisms of action in COVID-19 vaccines.**								
Totally disagree	1782	98.8	7	0.4	15	0.8	1804	100.0
Rather disagree	1540	97.5	29	1.8	10	0.6	1579	100.0
Neither agree nor disagree	437	86.0	42	8.3	29	5.7	508	100.0
Rather agree	269	71.0	61	16.1	49	12.9	379	100.0
Totally agree	78	38.0	25	12.2	102	49.8	205	100.0
Missing / no answer							25	
**The development and approval of COVID-19 vaccines are moving too fast for me.**								
Totally disagree	1792	99.4	5	0.3	6	0.3	1803	100.0
Rather disagree	1403	98.6	14	1.0	6	0.4	1423	100.0
Neither agree nor disagree	519	92.2	35	6.2	9	1.6	563	100.0
Rather agree	288	74.8	64	16.6	33	8.6	385	100.0
Totally agree	108	34.8	49	15.8	153	49.4	310	100.0
Missing / no answer							16	
**I generally trust the COVID-19 vaccines.**								
Totally disagree	46	27.7	11	6.6	109	65.7	166	100.0
Rather disagree	114	49.1	54	23.3	64	27.6	232	100.0

Neither agree nor disagree	358	79.7	70	15.6	21	4.7	449	100.0
Rather agree	1830	98.3	24	1.3	7	0.4	1861	100.0
Totally agree	1767	99.4	6	0.3	5	0.3	1778	100.0
Missing / no answer							14	
**I believe that the pharmaceutical industry cares more about its profit motives than safety of COVID-19 vaccines**								
Totally disagree	1020	98.3	8	0.8	10	1.0	1038	100.0
Rather disagree	1758	96.8	38	2.1	20	1.1	1816	100.0
Neither agree nor disagree	911	92.1	50	5.1	28	2.8	989	100.0
Rather agree	320	78.6	41	10.1	46	11.3	407	100.0
Totally agree	75	37.1	27	13.4	100	49.5	202	100.0
Missing / no answer							48	
**I trust the European regulatory authorities of COVID-19 vaccines used in Germany.**								
Totally disagree	38	30.9	6	4.9	79	64.2	123	100.0
Rather disagree	103	51.8	35	17.6	61	30.7	199	100.0
Neither agree nor disagree	299	74.2	65	16.1	39	9.7	403	100.0
Rather agree	1806	96.1	51	2.7	22	1.2	1879	100.0
Totally agree	1872	99.4	8	0.4	4	0.2	1884	100.0
Missing / no answer							12	
**I generally trust in German health care politics**								
Totally disagree	130	57.3	19	8.4	78	34.4	227	100.0
Rather disagree	426	79.6	49	9.2	60	11.2	535	100.0
Neither agree nor disagree	888	91.5	44	4.5	38	3.9	970	100.0
Rather agree	1835	96.3	45	2.4	26	1.4	1906	100.0
Totally agree	826	98.3	8	1.0	6	0.7	840	100.0
Missing / no answer							22	
**I generally trust in the medical opinion of physicians**								
Totally disagree	15	46.9	1	3.1	16	50.0	32	100.0
Rather disagree	98	74.2	12	9.1	22	16.7	132	100.0
Neither agree nor disagree	480	83.0	38	6.6	60	10.4	578	100.0
Rather agree	2078	92.4	88	3.9	83	3.7	2249	100.0
Totally agree	1438	96.4	27	1.8	26	1.7	1491	100.0
Missing / no answer							18	
**Number of correct answers in knowledge test**								
0	63	67.0	12	12.8	19	20.2	94	100.0
1	117	73.6	18	11.3	24	15.1	159	100.0
2	485	86.5	29	5.2	47	8.4	561	100.0
3	1100	92.2	51	4.3	42	3.5	1193	100.0
4	2160	97.1	33	1.5	31	1.4	2224	100.0
Missing / no answer							269	
**My primary care physician has given me …**								
advise to get vaccinated against COVID-19	500	96.5	8	1.5	10	1.9	518	100.0
advise not to get vaccinated against COVID-19	11	26.8	7	17.1	23	56.1	41	100.0
We have not discussed the vaccination so far	2019	90.9	98	4.4	103	4.6	2220	100.0

We have addressed the vaccination; my primary care physician has not made a recommendation for or against the vaccination	54	72.0	12	16.0	9	12.0	75	100.0
Missing / no answer							1646	
**The majority of my colleagues have / would like to …**								
get vaccinated against COVID-19	3486	96.8	62	1.7	54	1.5	3602	100.0
not get vaccinated against COVID-19	67	38.7	15	8.7	91	52.6	173	100.0
not yet decided whether to get vaccinated	293	74.6	68	17.3	32	8.1	393	100.0
we have not discussed vaccination so far	125	81.2	13	8.4	16	10.4	154	100.0
Missing / no answer							178	
**The majority of my family / friends have or would like to …**								
get vaccinated against COVID-19	3623	98.4	40	1.1	19	0.5	3682	100.0
not get vaccinated against COVID-19	68	27.1	31	12.4	152	60.6	251	100.0
not yet decided whether to get vaccinated	349	75.1	88	18.9	28	6.0	465	100.0
we have not discussed vaccination so far	57	85.1	5	7.5	5	7.5	67	100.0
Missing / no answer							35	
**From what media do you get your information in COVID-19 vaccination?**								
**Daily newspapers, weekly magazines (print)**								
Not selected	2984	91.2	121	3.7	168	5.1	3273	100.0
Selected	1141	93.0	46	3.7	40	3.3	1227	100.0
**Daily newspapers, weekly magazines (online)**								
Not selected	1731	88.9	89	4.6	127	6.5	1947	100.0
Selected	2394	93.8	78	3.1	81	3.2	2553	100.0
**Scientific journals (print)**								
Not selected	3357	91.8	138	3.8	161	4.4	3656	100.0
Selected	768	91.0	29	3.4	47	5.6	844	100.0
**Scientific journals (online)**								
Not selected	1664	91.3	78	4.3	81	4.4	1823	100.0
Selected	2461	91.9	89	3.3	127	4.7	2677	100.0
**Television / radio**								
Not selected	1618	89.0	69	3.8	130	7.2	1817	100.0
Selected	2507	93.4	98	3.7	78	2.9	2683	100.0
**Social networks (e.g., Facebook)**								
Not selected	3511	92.0	135	3.5	170	4.5	3816	100.0
Selected	614	89.8	32	4.7	38	5.6	684	100.0
**Messenger services (e.g., Twitter, Telegram)**								
Not selected	3943	92.4	155	3.6	169	4.0	4267	100.0
Selected	182	78.1	12	5.2	39	16.7	233	100.0

**Online video platforms (e.g., YouTube)**								
Not selected	3624	92.7	142	3.6	144	3.7	3910	100.0
Selected	501	84.9	25	4.2	64	10.8	590	100.0
**Websites / information portals of government health authorities (e.g., RKI, WHO)**								
Not selected	989	87.9	56	5.0	80	7.1	1125	100.0
Selected	3136	92.9	111	3.3	128	3.8	3375	100.0
**Other**								
Not selected	3732	91.7	158	3.9	179	4.4	4069	100.0
Selected	393	91.2	9	2.1	29	6.7	431	100.0

Supplemental Table S6: Attitudes and experiences with vaccinations, Odds ratios with corresponding 95% confidence intervals. Large values indicate higher odds for willingness to vaccinate. If not stated otherwise "neither agree nor disagree" was used as reference.

Predictor	Odds ratio	95% confidence interval<br>Lower	Upper
I trust the regulatory authorities of vaccines in Germany in general.			
Totally disagree	0.42	0.25	0.70
Rather disagree	0.37	0.24	0.58
Rather agree	5.64	4.13	7.70
Totally agree	47.15	29.76	74.72
I trust the European regulatory authorities of COVID-19 vaccines used in Germany.			
Totally disagree	0.16	0.10	0.24
Rather disagree	0.37	0.26	0.53
Rather agree	8.61	6.23	11.89
Totally agree	54.26	29.49	99.85
I basically trust in the German health care politics.			
Totally disagree	0.12	0.09	0.18
Rather disagree	0.36	0.26	0.49
Rather agree	2.39	1.72	3.31
Totally agree	5.45	3.07	9.68
I generally trust in the medical opinion of physicians.			
Totally disagree	0.18	0.09	0.37
Rather disagree	0.59	0.38	0.92
Rather agree	2.48	1.90	3.24
Totally agree	5.54	3.90	7.86
I already had a serious vaccine adverse effect that required medical treatment. (Ref: No)			
Yes	0.29	0.19	0.44
I make sure to keep my vaccinations up to date.			
Totally disagree	0.10	0.05	0.21
Rather disagree	0.66	0.38	1.16
Rather agree	1.97	1.22	3.16
Totally agree	4.57	2.87	7.29
I receive flu (influenza) vaccinations regularly.			
Totally disagree	0.25	0.16	0.40
Rather disagree	0.86	0.50	1.47
Rather agree	1.92	1.10	3.37
Totally agree	3.39	2.00	5.73
I feel well informed about COVID-19 vaccines.			
Totally disagree	0.08	0.05	0.13
Rather disagree	0.33	0.23	0.47
Rather agree	3.40	2.43	4.77
Totally agree	6.49	4.38	9.61
I believe the COVID-19 vaccines are effective.			
Totally disagree	0.21	0.12	0.35
Rather disagree	0.29	0.18	0.47
Rather agree	18.40	13.16	25.73
Totally agree	66.69	42.05	105.76
I generally trust COVID-19 vaccines.			
Totally disagree	0.10	0.06	0.15
Rather disagree	0.25	0.17	0.35
Rather agree	15.01	9.83	22.91
Totally agree	40.83	21.62	77.12
I believe the pharmaceutical industry cares more about its profit motives than safety of COVID-19 vaccines.			
Totally disagree	4.85	2.88	8.16
Rather disagree	2.60	1.83	3.68
Rather agree	0.31	0.23	0.44

Totally agree	0.05	0.04	0.07
I am afraid of short-term adverse effects of COVID-19 vaccines.			
Totally disagree	2.71	1.86	3.95
Rather disagree	2.03	1.43	2.89
Rather agree	0.46	0.32	0.67
Totally agree	0.14	0.09	0.21
I am afraid of long-term adverse effects from COVID-19 vaccines.			
Totally disagree	6.34	3.42	11.73
Rather disagree	5.19	2.96	9.09
Rather agree	0.24	0.16	0.36
Totally agree	0.04	0.03	0.07
I am concerned about what I consider novel mechanisms of action in COVID-19 vaccines.			
Totally disagree	13.16	8.07	21.47
Rather disagree	6.42	4.28	9.62
Rather agree	0.40	0.28	0.56
Totally agree	0.10	0.07	0.15
The development and approval of COVID-19 vaccines are moving too fast for me.			
Totally disagree	13.81	7.08	26.93
Rather disagree	5.95	3.47	10.19
Rather agree	0.25	0.17	0.37
Totally agree	0.05	0.03	0.07
I feel well informed about vaccines in general.			
Totally disagree	0.24	0.13	0.43
Rather disagree	0.55	0.37	0.83
Rather agree	2.82	2.05	3.87
Totally agree	4.40	3.17	6.09
I think vaccinations are generally effective			
Totally disagree	3.10	1.43	6.74
Rather disagree	0.61	0.28	1.35
Rather agree	6.05	3.64	10.06
Totally agree	34.21	20.91	55.98
I trust vaccines in general.			
Totally disagree	0.27	0.14	0.52
Rather disagree	0.36	0.21	0.62
Rather agree	4.54	3.14	6.57
Totally agree	21.02	14.25	31.01
I am generally afraid of adverse effects of vaccinations.			
Totally disagree	6.57	4.57	9.44
Rather disagree	4.03	2.87	5.64
Rather agree	0.82	0.56	1.20
Totally agree	0.41	0.27	0.62
I believe that the pharmaceutical industry's puts profit motives over the safety of vaccines.			
Totally disagree	3.04	1.98	4.65
Rather disagree	3.38	2.37	4.82
Rather agree	0.37	0.27	0.50
Totally agree	0.09	0.07	0.13
